# Supervised and Unsupervised Learning Technology in the Study of Rodent Behavior

**DOI:** 10.3389/fnbeh.2017.00141

**Published:** 2017-07-28

**Authors:** Katsiaryna V. Gris, Jean-Philippe Coutu, Denis Gris

**Affiliations:** Gris Lab of Neuroimmunology, Pediatrics, University of Sherbrooke Sherbrooke, QC, Canada

**Keywords:** animal behavior, automatic analysis, computer learning, supervised, unsupervised

## Abstract

Quantifying behavior is a challenge for scientists studying neuroscience, ethology, psychology, pathology, etc. Until now, behavior was mostly considered as qualitative descriptions of postures or labor intensive counting of bouts of individual movements. Many prominent behavioral scientists conducted studies describing postures of mice and rats, depicting step by step eating, grooming, courting, and other behaviors. Automated video assessment technologies permit scientists to quantify daily behavioral patterns/routines, social interactions, and postural changes in an unbiased manner. Here, we extensively reviewed published research on the topic of the structural blocks of behavior and proposed a structure of behavior based on the latest publications. We discuss the importance of defining a clear structure of behavior to allow professionals to write viable algorithms. We presented a discussion of technologies that are used in automated video assessment of behavior in mice and rats. We considered advantages and limitations of supervised and unsupervised learning. We presented the latest scientific discoveries that were made using automated video assessment. In conclusion, we proposed that the automated quantitative approach to evaluating animal behavior is the future of understanding the effect of brain signaling, pathologies, genetic content, and environment on behavior.

## Introduction

Rodent behavioral assessment tools that are implemented in research are lagging behind technological advancements in the computerized motion and feature recognition, statistical programing, and meta-data analysis. Annually, only a handful of publications utilize automated behavioral assessment approaches in the fields studying knock-out mice (Jhuang et al., 2010; de Chaumont et al., 2012; Kyzar et al., 2012; Ferhat et al., 2016), mouse disease models (Steele et al., 2007; Lee et al., 2016), drug testing in mice (Brodkin et al., 2014) and rats (Dunne et al., 2007), and social interactions (de Chaumont et al., 2012; Lo et al., 2016). It is widely recognized that automated video assessment exceeds any human manual assessment (Dunne et al., 2007; Schaefer and Claridge-Chang, 2012; Kabra et al., 2013; Dell et al., 2014; Egnor and Branson, 2016); yet due to its complexity, lack of appropriate training, and lack of resources, these superior technologies are not commonplace in research. In this review, we describe the structure of behavior and its constituents as it relates to computerized video assessment. We compare video-based supervised and unsupervised learning paradigms in automated behavioral assessment systems; their advantages and limitations. In conclusion, we highlight the importance of implementing automated behavioral assessment software in assessing rodent behavior in animal models of human diseases, drug testing, and other.

### Structure of behavior

A clear structure of behavior is needed to enable professionals to write viable algorithms, which can adequately evaluate such a multilayered subject. Over the past century, several behavioral researchers and ethologists have studied this subject in-depth. In 1953 in *The Study of Instinct*, Tinbergen warned that behavior should not be assessed as a fragment, but instead, should be considered in broad perspective to see each problem as a part of a whole. This is what the new technologies are aiming to achieve.

In 2014, Anderson and Perona co-authored a perspective publication in which they broke down behavior into building blocks: ethograms/activities, actions, and movements (Anderson and Perona, 2014). Through numerous examples working with drosophilae, authors used this structure to describe and explain organism's behavior using automated assessment tracking technologies. According to Anderson and Perona, movements are the simplest identifiable movements or trajectories such as step, rear-up, turn, etc. Actions are combinations of movements that occur in the same stereotypical sequence; for example, eat, walk, and assess threat. Actions and movements, in turn, are built into activities and can have stereotyped or variable structures, examples include aggression, parenting, and courtship (Anderson and Perona, 2014).

Gomez-Marin et al. in their 2014 review brought forward a possibility of establishing an ethome, defined as “a complete description of the set of behaviors manifested by species in their natural environment.” They propose that this ethome would vary in different environments and, as such, would have sub-ethomes, defined as the set of ethograms within a defined environment. He goes on to propose that with the improvements in big data analysis, we might be able to narrow down and identify fundamental behavioral units (ethons) (Gomez-Marin et al., 2014).

Similar structures were referred to in research papers of Wiltschko et al. (2015) and Vogelstein et al. (2014) with minor variations in terminology. Wiltschko et al. studying mouse behavior, call the simpler units of behavior, modules. They state that these reused and stereotyped modules change with a defined transition probability, which generates module sequences. Vogelstein et al. call the smaller units of behavior of *drosophila* larvae, behaviortypes. Further, they create a *Behaviortype Family Tree*, which gives sequential structure to behavior from simple movements to complex behavioral activities.

**Figure 1:**
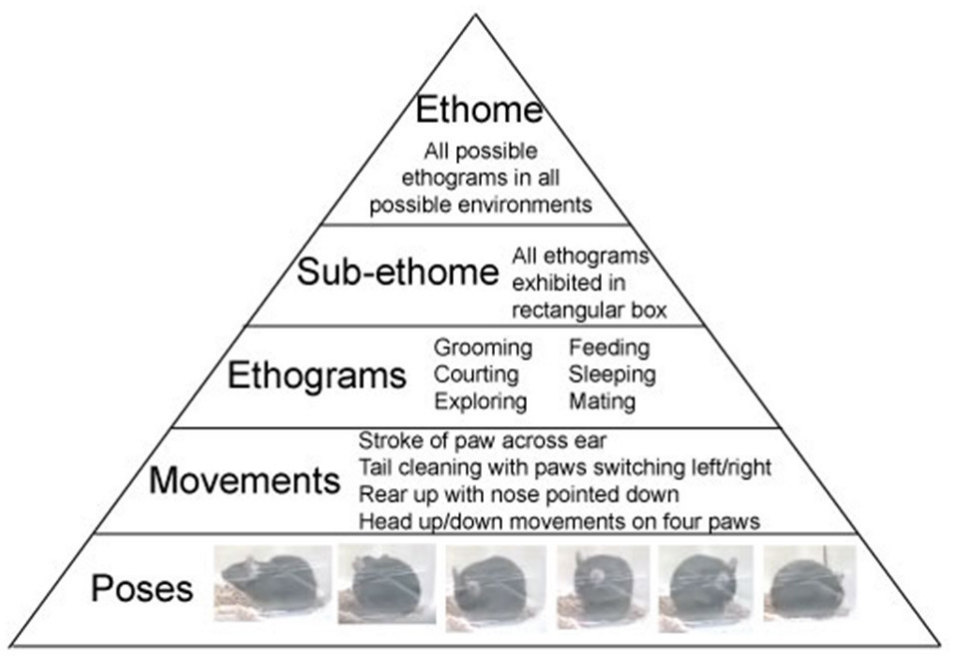
Structure of Behavior. Ethome of an animal consists of numerous sub-ethomes (a complete set of ethograms in a specified environment), which consist of ethograms (sets of repeatable, predefined, trainable, or innate movements), which consist of movements (the smallest complete motion). Movements are built from poses (postural snapshots in time). Examples of mouse behavior are presented in the pyramid.

The above structure of behavior is not novel, and it was discussed by the forefathers of ethology: Oskar Heinroth, Konrad Lorenz, and Niko Tinbergen. Using the computer vision technologies allows to observe individual behaviors in a continues sequence. Behavior is organized in a pyramid-like structure with, a multitude of poses at the bottom. Poses comprise movements in the second layer of the pyramid. In turn, movements build repeated sequences, called ethograms. Ethograms limited to a specific environment constitute sub-ethomes. And finally, all the sub-ethomes of an organism comprise its ultimate ethome (Figure 1).

### Ethograms

Ethograms are defined as repeatable and predefined sets of movements and they can be acquired or innate. Ethograms continuously change, get interrupted, and repeat themselves depending on the internal and/or external stimuli (Jones et al., 2016). In rodents, examples include grooming, nursing, courting, foraging, etc. In his recent work, Wiltschko et al. have quantified 65 ethograms (or modules) that were detected in an actively behaving mouse in a round arena (sub-ethome; Wiltschko et al., 2015) Over the past century, ethograms have been detected using traditional observations by experienced researchers (Kalueff and Tuohimaa, 2004; Han et al., 2017), by automated video assessment approaches (Wiltschko et al., 2015), and vibrations read-outs (Lutter et al., 2016).

### Movements

In turn, ethograms are comprised of movements, which are the smallest complete motion in a behaving animal such as step, turn, chew, rear-up, etc. (Anderson and Perona, 2014; Wiltschko et al., 2015). Numerous articles were published dissecting the paw movements in rats in reaching for food (Whishaw et al., 2003) and rat/mouse gait analysis (Ferber et al., 2016) using manual analysis of video recordings. Whishaw et al. described in detail using end-point measures (success vs. failure), kinematics (Cartesian representation of action, distance, velocity, and trajectories), movement description (Eshkol Wachman Movement Notation) the exact reaching and grabbing movements of a rat's forelimb use (Whishaw et al., 2008). These manual assessment techniques are extremely labor-intensive. They laid the foundation for understanding the relationship between brain and behavior, and provided the foundation for the first-generation of automated video assessment software. Today, automated assessment software simplifies and speeds up identification and quantification of these smaller movements (Kalueff and Tuohimaa, 2004; Kyzar et al., 2012).

### Poses

In turn, movements are comprised of poses, which are postural snapshots in time. Number of poses is indefinite considering that the snapshot can be taken at any point in time. The ability to break down behavior into poses allows a researcher to identify the cause for the change in the resulting ethogram.

### Automated behavioral assessment systems

The release of OpenCV (open source computer vision), a free computer vision library, in 2,000 has allowed for numerous developments in movement and object recognition technologies (Schaefer and Claridge-Chang, 2012). The availability of analytical programs, such as MatLab and R provide the tools needed to group and analyze the large amounts of behavioral data extracted in experiments (Colomb et al., 2012; de Chaumont et al., 2012; Gomez-Marin et al., 2012; Patel et al., 2014; Wiltschko et al., 2015). These technologies speed up the behavioral data collection and analysis with increasing accuracy, depth, and repeatability of experiments across laboratories (Spruijt et al., 2014). The real-time behavior analysis opened doors to correlative analysis of neuronal activation (optogenetics) to behavior phenotypes and ultrasonic vocalization to behavior phenotypes (Vogelstein et al., 2014; Ferhat et al., 2016; Han et al., 2017). In addition, automated assessment of video recordings allows for assessment of long-term experiments that last over 24 h. It was demonstrated that long-term behavioral experiments yield more reliable results compared to short-term (<15 min) assessments due to the habituation-length differences between mice strains and other factors (Fonio et al., 2012; Spruijt et al., 2014).

Automated behavioral assessment technologies have been used extensively as an aid in counting numbers of beams crossed, nose peaks, time spent, and distance traveled in mazes or open fields. In all these tests, the aid of the automation saved time and increased accuracy. However, here we are looking at the technology capable of analyzing and interpreting behavior of unrestraint rodents. Analyzing rodents in their home cages requires less handling; and, as such, causes less stress and anxiety in animals, increasing the repeatability of the experiments (Martin et al., 2014; Sorge et al., 2014; Spruijt et al., 2014).

As of today, most of the automated video behavioral analysis systems are still not plug and play. A certain level of understanding of the software is essential for ensuring correct interpretations of the results (Egnor and Branson, 2016). In this review, we are looking at systems that analyze unrestraint rodents in an unchallenged home cage environment. It is argued that challenging conditions must be provided for the rodents to extract behaviors that might not be detectable in the usual laboratory mouse cage. A review by Spruijt and DeVisser provides an in-depth discussion with examples of available “intelligent” home cages for such assessments (Spruijt and DeVisser, 2006).

### Supervised and unsupervised learning

Automated analysis software can be separated into two groups based on the learning paradigm: supervised and unsupervised. To create a software based on supervised learning, information about rodent behavior is taught to computers using specific instances of movement, which is assigned a name (as defined by researcher; Egnor and Branson, 2016). For example, various samples are taken of a mouse rearing. These photos are taught to computer software; and “rearing” is assigned to the movement. Therefore, the interpretation of the observed movements rests solely with the researcher. This approach utilizes the decades of work that was published by many laboratories around the world, which describe animal postures, movements, and ethograms. Movements are correlated to each other to create ethograms such as eating, grooming, foraging, etc. using Hidden Markov Model or other models (Stern et al., 2015). For example, in a home cage environment, an animal is rearing-up, if within a specified time it crosses a feeding box line, the algorithm regresses back to the already assigned “rear-up” behavior and changes it to eating.

In unsupervised learning software, the videos are fed into the computer software and a deep computer learning algorithm, such as neural networks or convoluted neural networks combines poses into categories based on mathematical annotation of required difference/similarity (Stern et al., 2015; Wiltschko et al., 2015; Egnor and Branson, 2016). Further, algorithms seek for repeatable sequences of poses, which become movements; and repeatable sequences of movements, which become ethograms. By placing an animal into various environments such as round arena, square home cage, etc., sub-ethomes can be established. The algorithms are written by a human and, as such, carry a certain bias, none-the-less the detection, compilation, and categorisation stays consistent and unbiased for all the animals in a study.

#### Supervised learning

Supervised learning software is used more often than the unsupervised learning (Brodkin et al., 2014; Dell et al., 2014). A number of open source software options are available for researchers, including Ctrax (http://ctrax.sourceforge.net/), Mice Profiler Tracker (http://icy.bioimageanalysis.org/plugin/) Mice_Profiler_Tracker, Sensory Orientation Software (SOS) track (https://sourceforge.net/projects/sos-track/), Autotyping (http://www.seas.upenn.edu/~molneuro/autotyping.html) and others. The five major behavioral software companies that sell ready-to-use solutions are Clever Sys, Noldus, TSE Systems, Biobserve, and HVS Image. Commercial software provides a user-friendly interface with various features including (but not limited to) analysis of home cage behavior, maze tracking, social interactions, open field test tracking, as well as an array of statistical analyses, which are automatically performed to provide the user with ready-to-use data sets. Unlike open source software, commercial packages do not require any prior programming training or knowledge. Although these programs are user friendly, there is a need for extensive tuning, adjustments, and customization of settings which is required to obtain reliable data. Human assessment of individual video segments is necessary for calibration of the analytical software and establishing the degree of accuracy of the analyses. Commercial software packages very costly, as they are priced separately for each individual module. Each module has limited technical specifications, and therefore, can produce only a narrow set of outputs for which it was designed. Software costs can rise rapidly since a researcher requires a number of modules to operate in tandem for any given project. Moreover, additional hardware that is proposed by the dealers is often advertised to improve the precision of analysis (i.e., cages, specific cameras, feeders, etc.) significantly contributes to the overall cost.

Despite prohibitive prices, the last decade saw several influential papers that focused on discovering novel and exciting aspects of behavior using these systems. A paper published in 2009 by Steele et al. used Clever Sys HomeCage software to improve phenotypic characterization of mouse models of Huntington's and prion disease, and has revealed earlier signs of onset of both diseases in mice. In prion disease, first phenotypic differences were detected using the software at 3.5 months post-injections compared to the standard 5.0–5.5 month post-injection (Steele et al., 2007).

Ferhat et al. used social scan software (Mice ProFiler) together with ultrasonic recordings to relate body movements to vocal deficits in mice missing *Shank2*, a gene associated with autism spectrum disorder. Using these technologies in synergy, Ferhat revealed that during male/female interactions, in the specific position of genital area sniffing, the vocalization of *Shank2* mice was significantly different from control animals (Ferhat et al., 2016).

### Unsupervised learning

Unsupervised learning approach is used less frequently, possibly because it is more novel and requires substantial computer programing skills that are not common among biologists. Scientific papers describing the first working models of unsupervised learning behavioral assessments were published in 2014–2015. As of today, there is no unsupervised learning software available on the market, neither open source nor commercial. A Harvard University laboratory with the leading author Wiltschko has developed an unsupervised learning software and published their first work characterizing mice behavior in various paradigms (Wiltschko et al., 2015).

Wiltschko's paper, published in 2015, shows that mouse behavior consists of short repeatable sequences (Wiltschko et al., 2015). They revealed that an actively behaving mouse moves every 350 ms on average; these unique individual movements are comprised into behavioral modules (or ethograms). The report defined 65 unique modules in an actively behaving mouse in a round arena, which, in turn, comprise 99% of all behavior exhibited by healthy C57Bl/6J male mice. This study utilized 3D imaging and autoregressive hidden Markov Model-based algorithm. Their system distinguished individual movements and deduced unique behavioral patterns based on the top-view video recordings of mice. Behavioral sequences were identified solely by the computer software, with the researcher assigning names to the sequences after the fact. They demonstrated that unsupervised learning software has a major benefit over supervised learning: it allows for the discovery of novel movements and recognition of deviations from usual movements that are not visible to human eye. It provides an unbiased reflection of the behaving animal (Wiltschko et al., 2015).

Another paper published in 2014, from Vogelstein et al. successfully incorporated unsupervised learning behavioral assessment analysis with optogenetics in order to trace the link between neuronal activation and behavior in *drosophila* larvae (Vogelstein et al., 2014). They used iterative denoising tree (IDT) methodology to generate analysis. *Drosophila* larvae are simpler creatures compared to mice, however Vogelstein et al. were able to confirm the previously known ethograms as well as uncover novel ones using this technology (Vogelstein et al., 2014).

### Limitations

The major limitation in the supervised learning is that the researcher must define movements and ethograms for the computer software. For example, there are many ways a mouse can rear-up, each is unique; yet, a researcher is unlikely to label each rearing variety differently and will simply combine them. Potentially, this may lead to misleading interpretation of the results. To address this issue, Clever Sys Inc. in their HomeCageScan software package separate rear-up movements into rear-up partially, rear-up full from partial, and direct rear-up from four paws. Even though this separation provides more details in the overall analysis of a behaving rodent, it is still not sensitive enough to point out differences in the actual posture.

Another important limitation in supervised learning stems from its inability to break down behavior into individual ethograms, movements, and poses. Usually, the software will provide the results as a mix of ethograms (grooming, eating) and movements (rearing, sniffing) (Steele et al., 2007). It is more appropriate to present results in ethograms; then upon request, a scientist should have access to the detailed movement information contained in each ethogram.

Even though supervised learning software can identify ethograms such as grooming and eating, it is not (yet) able to detect the beginning and the end of each individual ethogram cycle. Most of the time, when a mouse grooms it repeats each complete cycle a number of times. Being able to quantify how many complete ethogram cycles are performed and whether the ethograms get interrupted in mid-cycle can lead to uncovering novel mechanisms that link brain pathology to particular behavioral circuitry.

Humans are not able to detect full complement of the ethograms in what often appears to be sporadic rodent behavior. As such, it is likely that the unsupervised learning software with the use of well written algorithms will be the leading technology in breakthrough discoveries in behavioral circuitry.

## Conclusion

Supervised learning technologies provide a timely aid to the scientific community to make assessment of behavior more accessible across a variety of fields studying animal models of diseases, immunology, neuroscience, etc. In biomedical sciences, pathophysiological testing investigates etiology of the diseases on a molecular level. However, the overall picture of any disease can be better understood taking into consideration behavioral changes that accompany any given condition. The ability to express behavior quantitatively, to study the links between changes in brain signaling and behavioral output, opens the door to a more complete understanding of animal models of human disease, better interpretation of drug testing results, and as such, improved drug discovery. Today, supervised learning software is a realistic option for many laboratories as the results are easy to understand and interpret and implementation of such technology does not require computer science education or experience.

On the other hand, there is no suitable unsupervised learning software options available for the scientific community, which do not require computer science background. The algorithms have been developed and successfully used in multiple studies, however implementation of such technology in a laboratory is beyond the reach of most. Yet, unsupervised learning behavioral assessment offers a superior option of evaluating behavior in rodents.

Implementing automated behavioral assessment technologies is becoming more common place. Yet, there are still many fields were extensive manual quantitative behavioral measurements are used to evaluate the connections between neuronal alterations (inflammation, genetic variations, demyelination, etc.) and the resulting behavioral output. Many neuroinflammatory diseases such as Parkinson's, Alzheimer's, etc. have corresponding models in laboratory rodents. However, the thorough collection, analysis, and interpretation of the behavioral data are lacking. These models were built based on the molecular-cellular findings, which are correlated to human diseases. The ability to efficiently quantify various ethograms and movements is a step toward a more complete understanding of physiological events and their effects on an organism.

## Future perspectives of big behavioral data analysis

Most of the publications in ethology and behavioral sciences quantify the changes of behavioral parameters as a simple deviation from the control values. In parallel, the same approach was used at the dawn of the gene microarrays. Over time, the analysis tools of gene microarrays have greatly evolved and instead of two-condition comparison, the change over multiple experimental conditions over time was introduced (Slonim and Yanai, 2009). The enormous amounts of gene microarray data are now interpreted using automatic ontological analysis tools that are often based on MatLab and R (Khatri and Draghici, 2005). The same tools must be developed in behavioral big data which is acquired by the automated video assessment software. Not only will these powerful tools allow to seek statistical significance between large arrays of behavioral data, they will also allow to study behavior as dynamical information, quantifying transitional probabilities (Wiltschko et al., 2015) Clever Sys software already provides behavioral matrix data, which represents behaviors as a sequence in time.

## Author contributions

JC initiated writing the review article while being a student at Gris Lab. JC proof read and edited the final version. KG continued to work on the review article when JC has left the lab and finished writing the article. DG participated in conceptualizing the article, proofreading, and editing the work on every step of the way.

### Conflict of interest statement

The authors declare that the research was conducted in the absence of any commercial or financial relationships that could be construed as a potential conflict of interest.
